# Phase-Specific Damage Tolerance of a Eutectic High Entropy Alloy

**DOI:** 10.3390/e25121604

**Published:** 2023-11-30

**Authors:** Shristy Jha, Rajiv S. Mishra, Sundeep Mukherjee

**Affiliations:** 1 Department of Materials Science and Engineering, University of North Texas, Denton, TX 76203, USA; 2 Advanced Materials and Manufacturing Processes Institute, University of North Texas, Denton, TX 76207, USA

**Keywords:** micro-cantilever bending, eutectic system, high entropy alloy

## Abstract

Phase-specific damage tolerance was investigated for the AlCoCrFeNi_2.1_ high entropy alloy with a lamellar microstructure of L1_2_ and B2 phases. A microcantilever bending technique was utilized with notches milled in each of the two phases as well as at the phase boundary. The L1_2_ phase exhibited superior bending strength, strain hardening, and plastic deformation, while the B2 phase showed limited damage tolerance during bending due to micro-crack formation. The dimensionalized stiffness (DS) of the L1_2_ phase cantilevers were relatively constant, indicating strain hardening followed by increase in stiffness at the later stages and, therefore, indicating plastic failure. In contrast, the B2 phase cantilevers showed a continuous drop in stiffness, indicating crack propagation. Distinct differences in micro-scale deformation mechanisms were reflected in post-compression fractography, with L1_2_-phase cantilevers showing typical characteristics of ductile failure, including the activation of multiple slip planes, shear lips at the notch edge, and tearing inside the notch versus quasi-cleavage fracture with cleavage facets and a river pattern on the fracture surface for the B2-phase cantilevers.

## 1. Introduction

Multiphase alloys with heterogeneous microstructures demonstrate excellent combination of strength and ductility, making them attractive for myriad structural applications [1,2,3]. Recently, eutectic high entropy alloys (HEAs) with multiple solid solution phases have been the subject of extensive research due to their promise of overcoming the strength-ductility tradeoff seen in metals and conventional alloys [4,5,6,7,8,9,10,11]. The AlCoCrFeNi_2.1_ alloy represents a canonical example of an eutectic HEA with the lamellar arrangement of CoCrFe-rich L1_2_ and AlNi-rich B2 phases [4], which exhibit an excellent combination of strength and ductility at room temperature as well as exceptional strain hardening behavior that is largely retained up to 700 °C. Different processing routes have been utilized for achieving a wide range of microstructural features, resulting in outstanding mechanical properties and excellent corrosion resistance in these alloys. The ease of attaining ultrafine microstructure in directly cast eutectic HEAs is uncommon in conventional alloys [12]. However, there is a limited understanding of the response of these alloys to complex loading states and the initiation of failure at microstructural length-scales [5,6,8,12,13]. There are reports of brittle failure in eutectic HEAs subjected to loading at dynamic strain rates, such as during ballistic impact [14]. Evaluating the response of these novel alloys to complex loading states and understanding their intrinsic fracture mechanisms will allow for the design of damage-tolerant microstructures for further pushing the envelope of applications.

Fracture toughness measurements are primarily geared towards understanding two different responses of materials: (i) intrinsic mechanisms, which pertain to the initiation of cracks, and (ii) extrinsic mechanisms, which are related to the propagation of cracks. The latter involves various mechanisms such as crack deflection, trapping, and bridging that contribute to impeding crack growth until the point of crack arrest [15]. All prior studies on fracture behavior in eutectic HEAs are focused on macroscopic scale deformation and post mortem analysis, which provide limited insights into intrinsic versus extrinsic mechanisms [5,6,10,11,16]. Focused ion beam (FIB) milling and micro-mechanical testing have proven to be valuable tools in investigating mechanical behavior and intrinsic mechanisms of fracture [17,18,19,20,21]. Here, we report on the intrinsic fracture behavior and damage tolerance of the L1_2_ and B2 phases in AlCoCrFeNi_2.1_ EHEA through the utilization of microcantilever beams with notches milled in each of the phases for the in-depth understanding of failure initiation in these alloys and facilitate the design of microstructures that lead to a superior strength–ductility combination.

## 2. Materials and Methods

**Figure 1:**
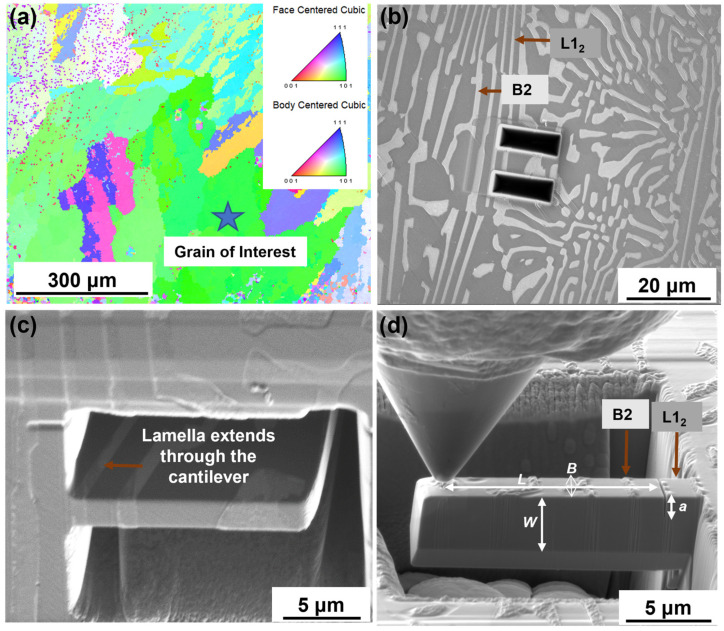
(**a**) Electron back-scattered diffraction micrograph showing an inverse pole figure of the region selected for milling the cantilevers; (**b**) secondary electron (SE) image of area selected to make the cantilever, showing a distinct phase color contrast between B2 and L1_2_; (**c**) SE image of the partially milled microcantilever cross-section, showing the B2 lamella extending through the thickness of the cantilever; (**d**) SE image of the fully milled microcantilever, showing the dimensions W, B, and L and also showing the alignment of the conical punch.

The AlCoCrFeNi_2.1_ EHEA was synthesized via vacuum-arc melting, wherein a predetermined quantity of the constituent pure elements was melted and homogenized. Following that, the samples underwent metallographic preparation for characterization, which involved mechanical grinding using SiC papers, with grit sizes ranging from 400 to 1200 and cloth polishing to obtain scratch free surfaces, using a 1 µm and 0.25 µm diamond solution. The final step in the preparation process involved the use of 0.02 μm colloidal silica to achieve a mirror finish. Microstructural characterization was carried out using FEI Nova Nano SEM230. The electron backscatter diffraction (EBSD) inverse pole figure (IPF) in Figure 1a shows the grain of interest. The selection of this grain was based on its size, which allowed for the milling of multiple microcantilevers without the influence of grain orientation on the properties. The secondary electron image in Figure 1b shows the lamellar arrangement of the B2 and the L1_2_ phases in the EHEA, each phase being 2–3 µm in width. The B2 phase shows lighter contrast and L1_2_ shows a darker contrast. The location of a representative micro-cantilever is also depicted in Figure 1b. To fabricate the microcantilevers, elongated and parallel lamellae were chosen for the milling of notches. As illustrated in Figure 1c, these lamellae extended through the thickness of the micro-cantilever, thereby enabling the notches to be entirely within the same phase.

The micro-cantilevers were fabricated using a focused ion beam (FIB, FEI Nova NanoLab 200) using Ga ions at an accelerating voltage of 30 keV. During the initial stages, a current of 20 nA was used, which was subsequently decreased to 100 pA for the final stage of polishing. The dimensions of the microcantilevers were consistently kept at 5 µm (B) × 5 µm (W) × 25 µm (L), where B represents the width, W represents the thickness, and L represents the distance between the notch and the point of loading as shown in Figure 1d. The notches were made in the L1_2_ phase, the B2 phase, and at their interface with the aim of measuring the phase-specific properties. The distance from the base of the cantilever was 3.5 µm, and a line pattern with a current of 10 pA was used to mill the notch, which resulted in the notch root radius being within 100 nm. The process outlined in reference [22] was utilized to fabricate the microcantilevers with a pentagonal cross-section and additional steps reported references [17,23,24] were considered during milling the notches. The dimensions of a representative microcantilever are shown in Figure 1d. The microcantilever bending experiments were conducted within the FIB-SEM using the Hysitron PI 88 Pico-indenter (Bruker, Minneapolis, MN, USA). A displacement-controlled mode was used with a 1 µm conical flat punch at 50 nm/s using a partial loading and unloading of up to 10% of the displacement. The maximum displacement was set at 8000 nm. The alignment of the probe with respect to the cantilever is depicted in Figure 1d. To ensure repeatability and measure the standard deviation, a minimum of five cantilevers were compressed for each phase.

The force versus displacement data obtained from the bending experiments were normalized to obtain bending stress (σ_b_) versus displacement curves using the relationship:

(1) $$\sigma_{b}\;=\mathrm{PLy}/I$$

where P is the load, L is the distance between the notch and the loading point, y is the distance between the top surface of the cantilever and the neutral plane in the vertical direction, and I is the moment of inertia for the pentagonal cross-section cantilever [22]. Further, the notched bending strengths of each phase was calculated using the relationship:

(2) $$\sigma_{\mathrm{nb}}\;=P_{\max}\mathrm{Ly}/I$$

where the value of P_max_ (taken as the maximum force attained during the bending) was obtained using Equation (1). As the dimensions do not satisfy the criteria given in ASTM E-399, the stress intensity factor is defined as the conditional stress intensity factor (K_Q_) and was calculated as [25]:

(3) KQ= σbπafaw

where $\sigma_{b}$ is the bending stress, a is the notch length, and f is a geometrical constant calculated for the pentagonal geometry as [22]:

(4) $$f\left(\frac{a}{W}\right)=1.85-3.38\left(\frac{a}{W}\right)+13.24{\left(\frac{a}{W}\right)}^{2}-23.26{\left(\frac{a}{W}\right)}^{3}+16.8{\left(\frac{a}{W}\right)}^{4}$$

Because the precise measurement of notch extension at every loading point in the loading-unloading load function was challenging, the overall conditional J integral was calculated as the sum of the elastic ($J_{\mathrm{el}}$) and plastic ($J_{\mathrm{pl}}$) contributions as below [26,27]:

(5) $$J_{C}={\;J}_{\mathrm{el}}+J_{\mathrm{pl}}=\frac{{\left(K_{\mathrm{QC}}\right)}^{2}{\left(1-\nu \right)}^{2}}{E}+\frac{{\eta A}_{\mathrm{pl}}}{B\left(W-a\right)+\frac{B^{2}}{4}}\;$$

where K_QC_ is the conditional critical stress intensity factor calculated using the value of $\sigma_{\mathrm{nb}}$, ν is the Poisson’s ratio of the material taken as 0.35, E is the elastic modulus calculated from the reduced modulus obtained from nano-indentation [28], η is a geometrical constant taken as 2, A_pl_ is the area under the plastic part of the loading curve calculated using Origin Pro, while B, W, and a are dimensions of the cantilever as shown in Figure 1d. The elastic–plastic stress intensity factor, (K_QJ_), was calculated using the following relation:

(6) $$K_{\mathrm{QJ}}=\surd \left(\frac{J_{C}E}{\left.{\left(1-\nu \right.}^{2}\right)}\right)$$

The diameter of the plastic zone size was estimated using the following relationship:

(7) $$D_{y}=10\ldots 50\frac{J}{\sigma_{y}}$$

where $\sigma_{y}$ is the yield strength that was calculated from micro-pillar compression in our previous work [28]. The dimensionalized stiffness (DS) values were calculated using the following equation [29]:

(8) $$\mathrm{DS}={\;\mathrm{kL}}^{3}/I$$

The stiffness values were obtained by fitting the unloading segment of the loading–unloading curves, while L and I represent the distance between the notch and the loading point and moment of inertia, respectively.

## 3. Results and Discussion

### 3.1. Phase-Specific Bending Response of the Microcantilevers

**Figure 2:**
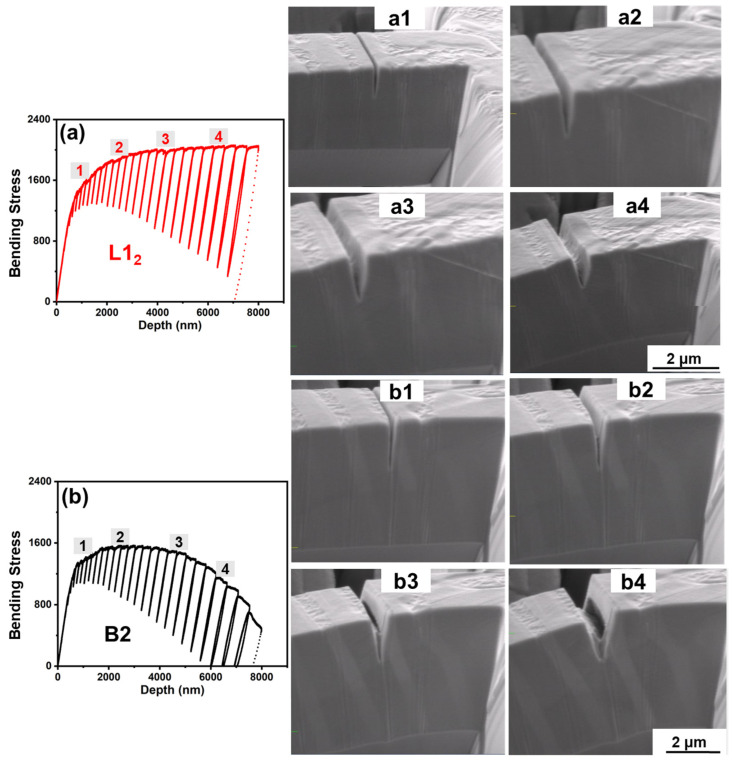
(**a**) Bending stress versus displacement curve of a representative cantilever with notch on the L1_2_ phase, with points 1, 2, 3, and 4 indicating different stages of deformation, and (**a1**–**a4**) associated with in situ SEM video shots; (**b**) bending stress vs. displacement curve of a representative cantilever with notch on the B2 phase with points 1, 2, 3, and 4, indicating different stages of deformation, and (**b1**–**b4**) associated in situ SEM shots.

Figure 2 shows the bending stress versus displacement curves and in situ SEM video shots of cantilevers with notches milled on the L1_2_ and B2 phases, respectively. The average bending strength of the cantilever with the notch on the L1_2_ phase (1980.1 ± 78.7 MPa) was ~40% higher than that with the notch on the B2 phase (1416.72 ± 228.7 MPa). The bending stress versus depth curves for the two cases show a distinct difference, as illustrated in Figure 2a,b. For the cantilever with the notch on the L1_2_ phase, the cantilever showed elastic response until point 1 followed by elastic–plastic deformation until point 2. This was followed by an increase in stress with deformation, indicating strain hardening in the material. Beyond point 3, the cantilever showed stable plastic deformation without a significant increase in stress. The corresponding in situ deformation images are shown in Figure 2(a1) with an undeformed notch, Figure 2(a2) with deformation primarily at the base of the cantilever and opening of the notch, Figure 2(a3) with further opening of the notch and tearing of the material, and finally, Figure 2(a4) with slip lines along the notch’s edge and tearing of the material. For the cantilever with notch on the B2 phase, the yield point was at a lower stress, and strain hardening was lesser compared to L1_2_ phase, as seen from the lower slope between points 1 and 2. After point 3, there was a continuous decrease in stress, indicating lower material resistance to deformation. The corresponding in situ deformation images presented in Figure 2(b1) displays the pristine, undeformed notch at point 1; Figure 2(b2) reveals the beginning of notch opening; Figure 2(b3) shows early indications of cavitation inside the notch at point 3; and finally, Figure 2(b4) shows significant cavitation growth corresponding to point 4, leading to further separation within the notch. The lighter contrast features at an angle on the cantilever surfaces seen in the SEM images of Figure 2(b1–b4) are artifacts from our FIB milling process. The two phases, L1_2_ and B2, exhibited different post-milling surface textures, namely a smoother finish for L1_2_ and a rougher surface texture for B2.

The extensive plastic deformation of the cantilever with the notch in the L1_2_ phase indicates that there was no void nucleation and crack growth in L1_2_ in contrast to the deformation seen in the B2 phase. The difference in notched cantilever bending behavior contrasts with what was found during the micropillar compression of the two phases [28]. The B2 phase had a ~32% higher compressive yield strength than the L1_2_ phase, which was attributed to nano-sized B2 precipitates hindering dislocation motion. However, such strengthening was not seen during the notched cantilever bending. Instead, catastrophic crack growth was observed. The initial work hardening prior to failure in Figure 2b indicates the beginning of plastic deformation, followed by cleavage fracture. The transition may be attributed to an abrupt sharpening of the crack radius, possibly due to stress concentration at the Cr-rich precipitates and multiaxial stress condition locally [30,31,32]. Cr-rich nanoprecipitates are reported to form in the B2 phase of AlCoCrFeNi_2.1_ systems as a result of spinodal decomposition driven by compositional modulation [11,33,34]. Nano-precipitates may contribute to the strengthening of an alloy by acting as dislocation inhibitors as well as dislocation generators at high stresses [35]. At the small scale, there may be limited contribution from intrinsic mechanisms such as dislocation interactions in the matrix due to lower material volume and the absence of extrinsic mechanisms to prevent crack propagation [15]. Hence, even though the Cr-rich precipitates helped initial strain hardening in the B2 phase, they did not inhibit crack propagation during the later stages of bending.

**Figure 3:**
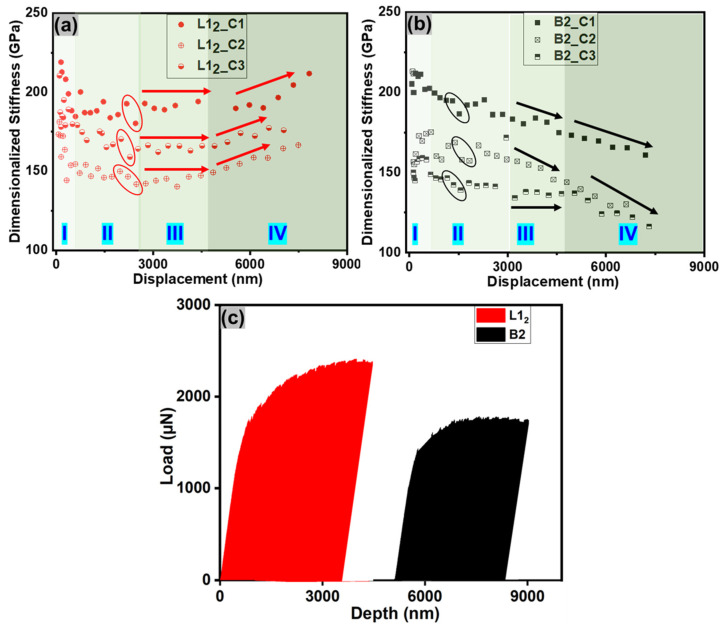
Dimensionalized stiffness (DS) for cantilevers with notches on the (**a**) L1_2_ and (**b**) B2 phases, as a function of displacement, showing four distinct stages of deformation during the micro-cantilever bending with arrows added as a guide to the eye; (**c**) area under the curves for calculating the energy dissipated until stage III of deformation for the two phases.

Figure 3a,b show the dimensionalized stiffness (DS) as a function of displacement for cantilevers with notches on the L1_2_ and B2 phases, respectively. These were calculated using the stiffness values generated from the unloading part of the curves until the maximum displacement of 8000 nm, as described in the experimental section. This represents variations in stiffness during cantilever deformation. The distribution may be separated into four distinct stages based on the bending curves given in Figure 2. The first stage represents elastic response until a displacement of 600–800 nm, a transition from elastic to plastic zone occurs between 800 nm and 2800 nm, a transition to plastic zone occurs between 2800 nm and 4300 nm, and the final stage, which represents failure. Three kinds of red and black symbols are used for three cantilevers with notches on the L1_2_ (Figure 3a) and B2 phases (Figure 3b), respectively. The stiffness distribution in the elastic stage was stochastic for both the phases, possibly due to the cantilever’s elastic spring-back due to very low loads [29,36]. The distribution was less stochastic in the second stage, during the transition from elastic to plastic zone, and both phases showed a similar trend of decline in the DS values. However, from the third stage onward, there were distinct differences between the two phases. The DS for L1_2_ was constant during stage III, followed by an increase in stage IV, likely due to strain hardening. In contrast, there was a continuous drop in stiffness of the B2 phase, indicating a decrease in resistance to the deformation response followed by crack growth.

The fracture toughness of ductile materials is calculated using the loading–unloading load function via the J integral approach (elastic–plastic fracture mechanics), where stiffness values are obtained during the test, which are derived using the unloading part of the curves. Because the energy required for the plastic deformation of a material depends on its stiffness, stiffness values were used to obtain the energy related to plastic deformation ahead of the crack tip [37,38]. In the case of a notch on the L1_2_ phase, the stiffness was constant during stage III, indicating dislocation interaction and leading to strain hardening and associated notch blunting, which is shown in Figure 2(a3). In stage IV, a rise in stiffness for L1_2_ indicates the plastic failure of the material. In the case of the B2 phase, an initial drop in DS value indicates crack nucleation followed by a sharp drop in stiffness suggesting subsequent crack growth (Figure 2(b4)). The area under the curves for the two cases is shown in Figure 3b, demonstrating the energy absorbed prior to failure in stage IV. The energy absorbed during cantilever deformation with a notch in L1_2_ (7.8 nJ) was 44% greater compared to the B2 phase (5.8 nJ). K_QJ_ was determined using Equation (6), using the values of energy absorbed for quantitative comparison between the two phases. K_QJ_ value obtained for the B2 phase was 15.54 ± 0.45 MPa$\sqrt{m}$, which is higher than the typical values for ideally brittle materials (~1–3 MPa$\sqrt{m}).$ The failure here may be considered as semi-brittle, which is typically characterized by irregular and complicated bifurcation, tunneling, and bridging zones [30,31,32]. As a result, the size of the fracture process zone varies substantially during crack propagation. Sample size-independent fracture toughness values are valid only when B and (W − a) > 10–50 J/$\sigma_{y}$. The ASTM standard E1820 defines $\sigma_{y}$ as the macroscopic yield strength, determined as the average of yield strength and ultimate tensile strength [39]. The plastic zone size in this case is close to or larger than the dimensions of the micro-cantilever. Therefore, the fracture toughness values obtained may not be size-independent. However, this approach provided valuable insights into the intrinsic crack propagation process, which highlighted the material’s fracture resistance facilitated by decohesion during small volume testing.

### 3.2. Post-Compression Analysis: Micro-Scale Fractography

**Figure 4:**
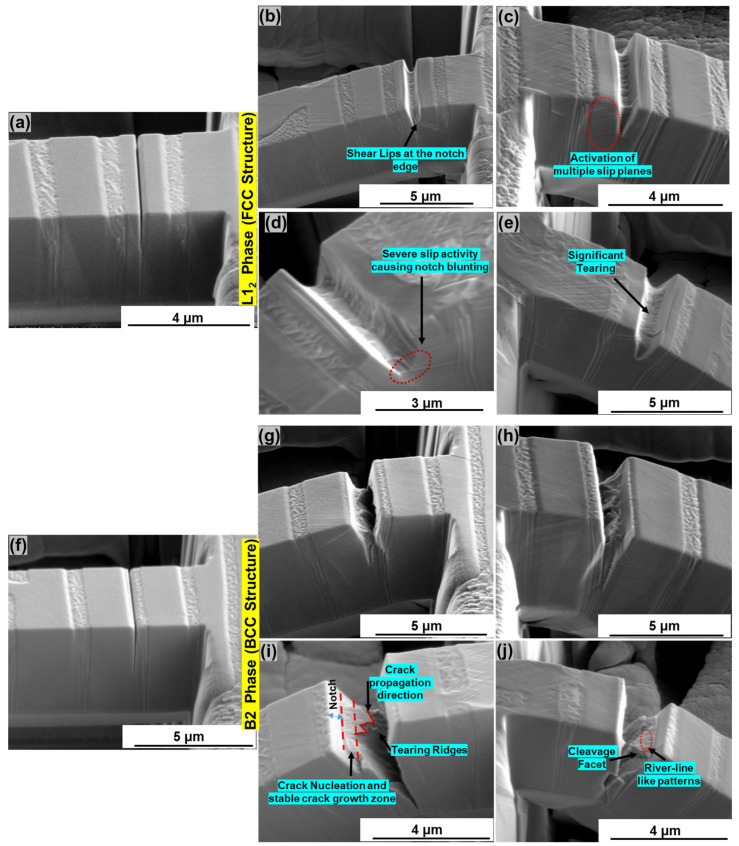
(**a**) SEM micrograph of a representative microcantilever showing the notch on L1_2_ phase; post-compression micrographs showing the deformation in and around the notch on the two sides of the cantilever compressed until (**b**,**c**) 8000 nm and (**d**,**e**) compressed to 16,000 nm; (**f**) SEM micrograph of a representative microcantilever showing the notch in the B2 phase; post-compression micrographs showing the deformation in and around the notch at both the sides of the same notch for the cantilever compressed until (**g**,**h**) 8000 nm and (**i**,**j**) further compressed to 16,000 nm.

Figure 4a shows an undeformed representative microcantilever with a notch on the L1_2_ phase. Figure 4b,c show the SE images of the two sides of the cantilever compressed to 8000 nm. Figure 4c shows the typical characteristics of ductile failure, including shear lips at the notch edge, tearing inside the notch, and the activation of several slip planes [30]. The cantilever, further compressed to 16,000 nm, showed the high density of shear steps at the notch edge, resulting in notch blunting and tearing, as shown in Figure 4d,e. Typical signs of ductile metal failure were observed, including shear steps at 45 degrees to the loading axis, significant plastic deformation supported by high slip activity, and notch blunting.

Figure 4f illustrates a typical cantilever with the notch on the B2 phase. Figure 4g,h shows the post-compression SE micrographs of the two sides of the cantilever compressed to 8000 nm. The presence of ridges suggests initial plastic deformation followed by rapid crack propagation. Figure 4i,j show typical signs of quasi-cleavage fracture upon further compression of the cantilever to 16,000 nm. The initial smooth zone near the surface of the notch indicates stable crack propagation. This was followed by cleavage facets and river pattern indicating rapid crack propagation and quasi-cleavage fracture. This form of failure is mainly caused by microscopic flaws such as microcracks or inclusions, leading to high stress-triaxiality and decohesion [31,32]. Microcracks in the B2 phase or Cr rich precipitates may have acted as points of stress triaxiality, resulting in sudden crack propagation rather than controlled growth by void coalescence. The Cr-rich precipitates in the B2 phase did not aid in dislocation strengthening by pinning, as previously demonstrated under tension and micro-pillar compression [6,11,16,28].

### 3.3. Bulk versus Microscale Fracture Mechanisms

The bulk uniaxial tensile deformation of AlCoCrFeNi_2.1_ EHEA showed extensive plasticity in L1_2_ phase and dislocation pile-up induced microcracks in the B2 phase, resulting in shear traces and fishbone-shaped crack marks. These microcracks eventually propagated along the B2–L1_2_ phase boundaries. Another study on the same alloy demonstrated considerable necking in the L1_2_ phase and several fracture modes in the B2 phase, including shear rib, cleavage, and tongue pattern [6,40,41]. The high strength of the B2 phase resulted in an overall increase in the strength of the alloy, and the back stresses resulted in a good strength–ductility combination. However, in this study, notched cantilever bending of the B2 phase showed quasi-cleavage mode of fracture. In bulk deformation, various extrinsic processes may constrain crack propagation. However, the absence of extrinsic mechanisms in the small-scale resulted in catastrophic failure of the B2 phase. Hence, quantifying phase-specific deformation mechanisms may aid in the design of the microstructure, such as the size and distribution of phases to achieve synergistic contribution. Secondly, bulk properties may not be scaled down to the micron-length scale, necessitating probing mechanical behavior at this scale when designing small-scale components, such as in microelectronics.

### 3.4. Microcantilever Bending with the Notch at the Interface

**Figure 5:**
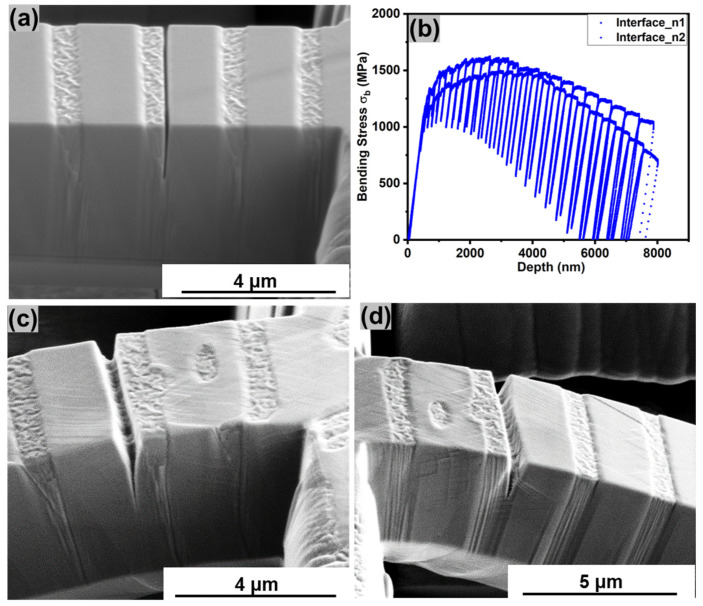
(**a**) SE micrograph of a cantilever with notch at the L1_2_–B2 interface; (**b**) bending stress vs. depth curves obtained from force-displacement data; (**c**,**d**) post-compression images showing deformation features around the notch.

Figure 5 shows the micro-cantilever bending analysis with the notch at the L1_2_–B2 interface. Figure 5a depicts a SE micrograph of a representative micro-cantilever with the notch at the interface. The bending stress versus displacement curves for the representative cantilevers are shown in Figure 5b. The bending response of cantilevers with a notch at the interface is intermediate between the two cases shown in the previous sections. It exhibited some strain hardening initially, like the L1_2_ case, but the slope of the curves dropped during the final stage of deformation, like the B2 case, indicating crack growth. However, the failure was not catastrophic and quasi-cleavage fracture was not seen, as is clear from the post-deformation micrographs in Figure 5c,d. This indicates that the interface was stable since there were no signs of decohesion [16,28].

## 4. Conclusions

The phase-specific damage tolerance of AlCoCrFeNi_2.1_ EHEA was studied, with lamellar microstructures consisting of L1_2_ and B2 phases. In situ microcantilever bending technique was used with notches milled in the two phases and at the phase boundary.

- The cantilever with the notch on the L1_2_ phase demonstrated superior bending strength and ductility over the B2 phase notched cantilever, which yielded lower stress and deformed less. This divergence from phase-specific micropillar compression behavior may be due to micro-crack formation in the B2 phase under bending stress and the reduced effectiveness of nano-precipitate strengthening due to a smaller test volume and a lack of extrinsic mechanisms to hinder crack growth.
- The bending deformation process showed four distinct stages based on dimensionalized stiffness (DS) values. L1_2_ and B2 phases showed significant stiffness variations, with L1_2_ cantilevers showing constant DS values due to strain hardening during stage III and increases in DS values indicating plastic failure during stage IV. B2 cantilevers showed an initial drop in DS during stage III (micro-crack formation) and a sharp drop in DS during stage IV, indicating crack propagation. The energy absorbed during deformation in the presence of a notch in L1_2_ phase was 44% higher than in B2 phase.
- The post-compression fractography of the L1_2_ phase showed signs of ductile failure, including shear lips in the notch and the tearing and activation of several slip planes. On the other hand, the B2 phase showed a quasi-cleavage fracture caused by microcracks or inclusions, which may be attributed to high stress-triaxiality and decohesion.
- Micro-cantilever bending with a notch at the L1_2_–B2 interface exhibited an intermediate bending response between cases where the notch was placed entirely in the L1_2_ and B2 phases, with some strain hardening indicating interface stability.

## Data Availability

Data are contained within the article.

## References

[1] Ma E., Wu X. (2019). Tailoring heterogeneities in high-entropy alloys to promote strength–ductility synergy. Nat. Commun..

[2] Shi P., Zhong Y., Li Y., Ren W., Zheng T., Shen Z., Yang B., Peng J., Hu P., Zhang Y. (2020). Multistage work hardening assisted by multi-type twinning in ultrafine-grained heterostructural eutectic high-entropy alloys. Mater. Today.

[3] Sauthoff G. (2000). Multiphase intermetallic alloys for structural applications. Intermetallics.

[4] Lu Y.P., Dong Y., Guo S., Jiang L., Kang H.J., Wang T.M., Wen B., Wang Z.J., Jie J.C., Cao Z.Q. (2014). A promising new class of high-temperature alloys: Eutectic high-entropy alloys. Sci. Rep..

[5] Wani I.S., Bhattacharjee T., Sheikh S., Lu Y.P., Chatterjee S., Bhattacharjee P.P., Guo S., Tsuji N. (2016). Ultrafine-grained AlCoCrFeNi2.1 eutectic high-entropy alloy. Mater. Res. Lett..

[6] Bhattacharjee T., Wani I.S., Sheikh S., Clark I.T., Okawa T., Guo S., Bhattacharjee P.P., Tsuji N. (2018). Simultaneous Strength-Ductility Enhancement of a Nano-Lamellar AlCoCrFeNi2.1 Eutectic High Entropy Alloy by Cryo-Rolling and Annealing. Sci. Rep..

[7] Wani I., Bhattacharjee T., Sheikh S., Bhattacharjee P., Guo S., Tsuji N. (2016). Tailoring nanostructures and mechanical properties of AlCoCrFeNi2.1 eutectic high entropy alloy using thermo-mechanical processing. Mater. Sci. Eng. A.

[8] Reddy S., Yoshida S., Sunkari U., Lozinko A., Joseph J., Saha R., Fabijanic D., Guo S., Bhattacharjee P., Tsuji N. (2019). Engineering heterogeneous microstructure by severe warm-rolling for enhancing strength-ductility synergy in eutectic high entropy alloys. Mater. Sci. Eng. A.

[9] Wang L., Yao C., Shen J., Zhang Y., Wang T., Ge Y., Gao L., Zhang G. (2020). Microstructures and room temperature tensile properties of as-cast and directionally solidified AlCoCrFeNi2.1 eutectic high-entropy alloy. Intermetallics.

[10] Wang T., Komarasamy M., Shukla S., Mishra R.S. (2018). Simultaneous enhancement of strength and ductility in an AlCoCrFeNi2.1 eutectic high-entropy alloy via friction stir processing. J. Alloys Compd..

[11] Gao X., Lu Y., Zhang B., Liang N., Wu G., Sha G., Liu J., Zhao Y. (2017). Microstructural origins of high strength and high ductility in an AlCoCrFeNi2.1 eutectic high-entropy alloy. Acta Mater..

[12] Lu Y., Dong Y., Jiang H., Wang Z., Cao Z., Guo S., Wang T., Li T., Liaw P.K. (2020). Promising properties and future trend of eutectic high entropy alloys. Scr. Mater..

[13] Huang L., Sun Y., Chen N., Luan H., Le G., Liu X., Ji Y., Lu Y., Liaw P.K., Yang X. (2022). Simultaneously enhanced strength-ductility of AlCoCrFeNi2.1 eutectic high-entropy alloy via additive manufacturing. Mater. Sci. Eng. A.

[14] Muskeri S., Jannotti P.A., Schuster B.E., Lloyd J.T., Mukherjee S. (2022). Ballistic impact response of complex concentrated alloys. Int. J. Impact Eng..

[15] Ritchie R.O. (2011). The conflicts between strength and toughness. Nat. Mater..

[16] Wang Q., Lu Y., Yu Q., Zhang Z. (2018). The Exceptional Strong Face-centered Cubic Phase and Semi-coherent Phase Boundary in a Eutectic Dual-phase High Entropy Alloy AlCoCrFeNi. Sci. Rep..

[17] Gabel S., Giese S., Merle B., Sprenger I., Heilmaier M., Neumeier S., Bitzek E., Göken M. (2021). Microcantilever Fracture Tests on Eutectic NiAl–Cr(Mo) In Situ Composites. Adv. Eng. Mater..

[18] Tian C., Kirchlechner C. (2021). The fracture toughness of martensite islands in dual-phase DP800 steel. J. Mater. Res..

[19] Gabel S., Giese S., Webler R.U., Neumeier S., Göken M. (2022). Microcantilever Fracture Tests of α-Cr Containing NiAl Bond Coats. Adv. Eng. Mater..

[20] Gabel S., Merle B., Bitzek E., Göken M. (2022). A new method for microscale cyclic crack growth characterization from notched microcantilevers and application to single crystalline tungsten and a metallic glass. J. Mater. Res..

[21] Ast J., Ghidelli M., Durst K., Göken M., Sebastiani M., Korsunsky A.M. (2019). A review of experimental approaches to fracture toughness evaluation at the micro-scale. Mater. Des..

[22] Di Maio D., Roberts S. (2005). Measuring fracture toughness of coatings using focused-ion-beam-machined microbeams. J. Mater. Res..

[23] Iqbal F., Ast J., Göken M., Durst K. (2012). In situ micro-cantilever tests to study fracture properties of NiAl single crystals. Acta Mater..

[24] Best J.P., Zechner J., Shorubalko I., Oboňa J.V., Wehrs J., Morstein M., Michler J. (2016). A comparison of three different notching ions for small-scale fracture toughness measurement. Scr. Mater..

[25] Documents R., Factor S., Toughness P.F. (1997). Standard Test Method for Plane-Strain Fracture Toughness of Metallic Materials 1. Configurations.

[26] Chu Q., Cao Q., Zhu X., Zhang M., Zhu Z., Zhang H., Bai R., Lei Z., Cheng P., Yan C. (2022). Fracture behavior and deformation-induced structure changes of a Ti-based metallic glass using micro-sized cantilevers. Mater. Sci. Eng. A.

[27] Sorensen D., Hintsala E., Stevick J., Pischlar J., Li B., Kiener D., Myers J.C., Jin H., Liu J., Stauffer D. (2020). Intrinsic toughness of the bulk-metallic glass Vitreloy 105 measured using micro-cantilever beams. Acta Mater..

[28] Muskeri S., Hasannaeimi V., Salloom R., Sadeghilaridjani M., Mukherjee S. (2020). Small-scale mechanical behavior of a eutectic high entropy alloy. Sci. Rep..

[29] Choi J.-H., Kim H., Kim J.-Y., Lim K.-H., Lee B.-C., Sim G.-D. (2022). Micro-cantilever bending tests for understanding size effect in gradient elasticity. Mater. Des..

[30] Pippan R., Wurster S., Kiener D. (2018). Fracture mechanics of micro samples: Fundamental considerations. Mater. Des..

[31] Chen J.-H., Cao R. (2014). Micromechanism of Cleavage Fracture of Metals: A Comprehensive Microphysical Model for Cleavage Cracking in Metals.

[32] Pineau A., Benzerga A.A., Pardoen T. (2016). Failure of metals I: Brittle and ductile fracture. Acta Mater..

[33] Charkhchian J., Zarei-Hanzaki A., Moshiri A., Abedi H.R., Schwarz T.M., Lawitzki R., Schmitz G., Chadha K., Aranas C., Shen J. (2023). Spinodal Decomposition of B2-phase and Formation of Cr-Rich Nano-precipitates in AlCoCrFeNi2.1 Eutectic High-Entropy Alloy. Adv. Eng. Mater..

[34] Borkar T., Gwalani B., Choudhuri D., Alam T., Mantri A., Gibson M., Banerjee R. (2016). Hierarchical multi-scale microstructural evolution in an as-cast Al2CuCrFeNi2 complex concentrated alloy. Intermetallics.

[35] Peng S., Wei Y., Gao H. (2020). Nanoscale precipitates as sustainable dislocation sources for enhanced ductility and high strength. Proc. Natl. Acad. Sci. USA.

[36] Li B.-S., Marrow T.J., Roberts S.G., Armstrong D.E.J. (2019). Evaluation of Fracture Toughness Measurements Using Chevron-Notched Silicon and Tungsten Microcantilevers. JOM.

[37] Zhu X.-K., Joyce J.A. (2012). Review of fracture toughness (G, K, J, CTOD, CTOA) testing and standardization. Eng. Fract. Mech..

[38] Ast J., Przybilla T., Maier V., Durst K., Göken M. (2014). Microcantilever bending experiments in NiAl—Evaluation, size effects, and crack tip plasticity. J. Mater. Res..

[39] (2001). Standard Test Method for Measurement of Fracture Toughness.

[40] Zheng H., Chen R., Qin G., Li X., Su Y., Ding H., Guo J., Fu H. (2019). Phase separation of AlCoCrFeNi2.1 eutectic high-entropy alloy during directional solidification and their effect on tensile properties. Intermetallics.

[41] Peng P., Li S., Chen W., Xu Y., Zhang X., Ma Z., Wang J. (2022). Phase selection and mechanical properties of directionally solidified AlCoCrFeNi2.1 eutectic high-entropy alloy. J. Alloys Compd..

